# Enzyme-Gelatin Electrochemical Biosensors: Scaling Down

**DOI:** 10.3390/bios2010101

**Published:** 2012-03-15

**Authors:** Karolien De Wael, Stijn De Belder, Sanaz Pilehvar, Geert Van Steenberge, Wouter Herrebout, Hendrik A. Heering

**Affiliations:** 1 Environmental Analysis, University of Antwerp, Universiteitsplein 1, B-2610 Antwerp, Belgium; E-Mails: stijn.debelder@ua.ac.be (S.D.B.); sanaz.pilehvar@ua.ac.be (S.P.); wouter.herrebout@ua.ac.be (W.H.); 2 Centre for Microsystems Technology, Department of Electronics and Information Systems, Ghent University-imec, Technologiepark 914A, B-9052 Ghent, Belgium; E-Mail: geert.vansteenberge@elis.ugent.be; 3 Leiden Institute of Chemistry, Leiden University, Einsteinweg 55, NL-2333 CC Leiden, The Netherlands; E-Mail: h.a.heering@chem.leidenuniv.nl

**Keywords:** gelatin, hydrogel, biosensor, spin coating, catalase, biocatalysis, hydrogen peroxide

## Abstract

In this article we investigate the possibility of scaling down enzyme-gelatin modified electrodes by spin coating the enzyme-gelatin layer. Special attention is given to the electrochemical behavior of the selected enzymes inside the gelatin matrix. A glassy carbon electrode was used as a substrate to immobilize, in the first instance, horse heart cytochrome *c* (HHC) in a gelatin matrix. Both a drop dried and a spin coated layer was prepared. On scaling down, a transition from diffusion controlled reactions towards adsorption controlled reactions is observed. Compared to a drop dried electrode, a spin coated electrode showed a more stable electrochemical behavior. Next to HHC, we also incorporated catalase in a spin coated gelatin matrix immobilized on a glassy carbon electrode. By spincoating, highly uniform sub micrometer layers of biocompatible matrices can be constructed. A full electrochemical study and characterization of the modified surfaces has been carried out. It was clear that in the case of catalase, gluteraldehyde addition was needed to prevent leaking of the catalase from the gelatin matrix.

## 1. Introduction

Recently, electrochemical biosensors have been developed for studying biological reactions and biologically active compounds which play an important role in food and drug analysis [1,2,3,4,5]. These sensors have superior properties such as simple measurement process, low cost, rapid response and increased sensitivity and selectivity. A biological element (enzyme or antibody, *etc*.) and a transducer are the two main components of biosensors. The monitoring of the interaction between the target analyte and the enzyme by a transducer results in a response which can be measured. So far, many attempts have been made in order to enhance transduction responses by immobilizing a bio recognizer element [6,7]. The activity of the bio-recognition elements should be maintained when the elements are immobilized on the electrode surfaces. Therefore, several approaches are envisaged to create a stable and active sensing device [8,9,10,11]. 

In general, different techniques have been used for the immobilization of biocatalysts: adsorption, ionic or covalent binding by using a chemical coupling agent, crosslinking, entrapment and encapsulation, protein fusion and enzymatic conjugation. Especially, covalent bonding and crosslinking methods are widely used in biosensor fabrication, because this promotes long operational performance [12,13]. Different materials have been used as suitable matrices for entrapment, such as agar, alginate, chitin, chitosan, agarose, cellulose, collagen, polyvinylalcohol, and gelatin [14,15]. Gelatin has received much consideration because it is an intrinsically biocompatible polymer [16,17,18]. It forms a hydrophilic network that is suitable for entrapment of biomolecules. As has been reported, horse heart cytochrome *c* and cytochrome *c* peroxidase can be incorporated successfully in a gelatin matrix [16,18]. On the other hand, gelatin may suffer from poor mechanical strength because of its ability to swell in an aqueous solution. The mechanical and thermal stability of the biopolymer can be promoted by crosslinking [17,19,20]. Glutaraldehyde (GTA) is widely used as a chemical crosslinking agent and is easily available and inexpensive. Crosslinking with GTA entails the reaction of free amino group of a polypeptide chain of gelatin with the aldehyde group of GTA [20]. In this work, gelatin is selected as matrix for the encapsulation of enzymes.

Sensitive and selective detection of hydrogen peroxide (H_2_O_2_) is necessary in many areas such as biomedical application and environmental chemistry [21,22]. The reduction of hydrogen peroxide is catalyzed by a large number of enzymes that are essential in food, pharmaceutical and clinical analyses. In addition, hydrogen peroxide is a reactive metabolic by-product that plays a key role in a number of oxidative stress-related processes. Among various common techniques for monitoring hydrogen peroxide such as spectrophotometry [23], fluorometry [24], and chemiluminescence [25], electrochemical biosensors (on which enzymes are immobilized) are more accurate, rapid and offer lower detection limits.

Catalase (Cat) is a common heme containing enzyme with four equal units having ferroprotoprophyrin at the centre. Its ability to catalyze the disproportionation of hydrogen peroxide into oxygen and water without the formation of free radicals (according to the reaction 1–1) makes catalase an attractive choice for the electrochemical determination of hydrogen peroxide [26,27,28,29].



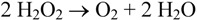
(1)

In this work, the heme containing protein catalase was immobilized in a gelatin film on a glassy carbon electrode surface. We report on the use of the spin coating method to prepare modified electrodes with continuous gelatin films in which, cytochrome *c* (as proof of principle) and catalase were encapsulated. By spincoating, highly uniform sub micrometer layers of biocompatible matrices can be constructed. This results in a more reproducible response compared to manual deposition techniques that lead to thick and asymmetrical layers [30,31].

## 2. Experimental Section

### 2.1. Chemicals and Solutions

Horse heart cytochrome *c* (HHC), catalase (Cat), 2-[4-(2-hydroxyethyl)-piperazinyl]ethanesulfonic acid (HEPES), sodium hydroxide, gluteraldehyde (Glu) were purchased from Sigma-Aldrich. The HEPES buffer solution of 10 × 10^−3^ mol∙L^−1^ was set to pH 7.0 using a 0.15 mol∙L^−1^ NaOH solution. Type B gelatin (Gel, IEP = 5, Bloom strength = 257), isolated from bovine skin by the alkaline process, was kindly supplied by Tessenderlo Chemie (Belgium). 

### 2.2. Electrode Preparation

The three-electrode system consists of a saturated calomel reference electrode (SCE, Radiometer Analytical, France), a graphite counter electrode and a glassy carbon inlaid disc electrode. The glassy carbon working electrodes of 3 mm diameter were pretreated by mechanical polishing. Before its first use the electrode surface was briefly polished in ethanol on an aluminum oxide film disc with 0.012 mm particles to obtain a smooth and clean surface. To remove any adherent Al_2_O_3_ particles the electrode surface was rinsed thoroughly with deionised water and dried with a tissue. Subsequently, the electrode was rubbed over a filter paper with ethanol, after which the electrodes were rinsed with water and left to dry at room temperature.

To immobilize a drop dried layer of gelatin B onto a glassy carbon electrode, 10 µL of a gelatin B solution (5% w/w)/HEPES mixture was placed on the surface by means of a pipette and was left to dry in air at 4 °C. We refer to these thick layers as drop dried (DD) layers in the text. To obtain a spin coated (SC) gelatin layer, a 10 µL drop was applied to the surface in the same way, but afterwards the electrode was rotated very briefly (5 s) at 1,500 rpm. 

The gelatin B solution was prepared by mixing the gelatin B powder and the HEPES buffer solution at approximately 40 °C, as was described earlier [16,18]. These electrodes are referred to in the text as GelB|GC. If a HHC or Cat solution is used instead of pure HEPES, the final electrodes are denoted as HHC|GelB|GC (3:7 ratio) or Cat|GelB|GC (3:7 ratio). The concentration of HHC, Cat and gelatin is 0.15 mM, 0.15 mM, 5 m% respectively unless stated otherwise. 

### 2.3. Apparatus

A µ-Autolab potentiostat controlled by GPES 4.9 007 software package (Metrohm, The Netherlands) was used for recording the cyclic voltammetric curves. The solutions were thoroughly deoxygenated by bubbling with nitrogen for at least 30 min, and again for approximately 5 min in the cell itself before usage.

The selected approach for determining the gelatin layer thickness is locally to remove the film with an Excimer laser and to measure the step height using a non-contact optical profiler (WYKO NT3300). The KrF Excimer laser (ATL Lasertechnik SP300i) operates at a wavelength of 248 nm, has a pulse length of 3–7 ns (FWHM), and for the experiments a pulse repetition rate of 100 Hz was used. A circular mask with an aperture of 2mm was applied to utilize a homogeneous part of the beam, which was focused by a lens onto the sample with a demagnification of 10, yielding a spot size of 200 μm. The fluence was adjusted with an attenuator plate, and the pulse energy was measured by a pyroelectric energy meter (Coherent J25LP-MUV, in combination with FieldmaxII TOP) placed at the end of the beam line. To fully remove the gelatin layer, 400 laser pulses were applied, using a laser fluence of 150 mJ/cm^2^.

Surface investigation of the prepared films was done on a JEOL JSM-5510 Scanning Electron Microscope (SEM). Samples were coated with a thin gold layer to improve electrical conductivity by means of a Balzers FL-9496 coater.

Infrared spectra at 4 cm^−1^ resolution was recorded using a Bruker Vector 22 FTIR spectrometer equipped with a PIKE Miracle single reflection ATR accessory.

## 3. Results and Discussion

### 3.1. Electrochemical Behaviour of HHC in Gelatin on Glassy Carbon Electrodes: Drop Dried (DD) *versus* Spin Coated (SC) Layers

**Figure 1:**
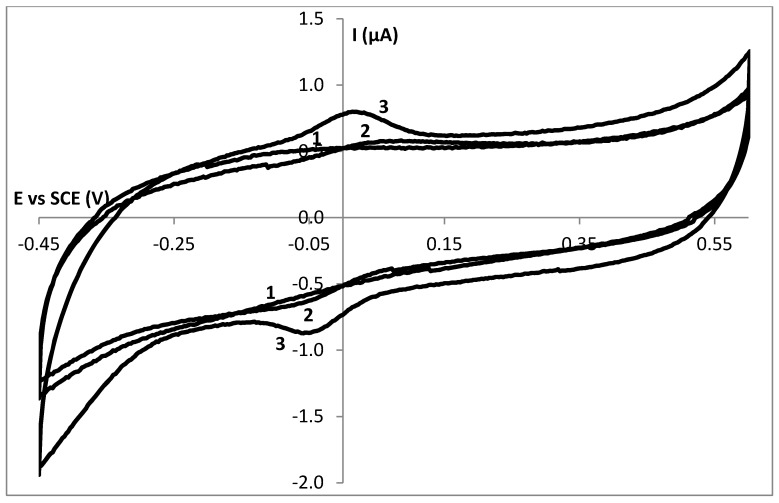
The current potential behaviour of a GelB|C (1), a drop dried HHC|GelB|C (2) and a spin coated HHC|GelB|C (3) electrode in a 10 mmol∙L^−1^ HEPES pH 7 buffer solution with a scan rate of 50 mV∙s^−1^.

Figure 1 shows the current-potential behavior of a drop dried (DD) GelB|GC electrode (1) and a DD HHC|GelB|GC electrode (2) in a 10 mmol∙L^−1^ HEPES pH 7 buffer solution in a potential window from −0.4 to 0.6 V with a scan rate of 50 mV∙s^−1^. No oxidation or reduction process is observed at a GelB|GC electrode. In this potential window, the gelatin matrix seems not to be electrochemically active at a glassy carbon electrode. When 0.5 mmol∙L^−1^ HHC is added to a GelB matrix, the corresponding current potential behavior is shown as curve 2 (which is the first scan obtained during the cyclic voltammetric experiment). The voltammogram shows a well-defined oxidation and reduction wave with peak potentials of 55 and −73 mV respectively, as expected for the oxidation and the reduction of the heme group present in the HHC protein [16,32,33]. The midpoint potential is 4 mV, consistent with the formal potential of HHC in solution [34]. In a previous article [16], the electrochemical behavior of a DD HHC|GelB|MH|Au electrode (MH = mercaptohexanol) was described. Similar diffusion controlled phenomena were observed. Within the thick DD gelatin layer, electron hopping between the biomolecules is responsible for the diffusion behavior [35].

The average surface concentration of DD HHC in gelatin is ca. 7 × 10^−11^ mol∙cm^−2^. Taking into account the initial total amount of proteins on the surface, the fraction of electroactive molecules is rather low, *i.e.*, two layers of cytochrome *c* molecules can be detected electrochemically [36]. The same phenomenon was described earlier [37]: while the concentrations inside the gel increase, the fraction of electroactive HHC decreased dramatically. This implies that only the molecules close to the surface are electrochemically active, as expected for the viscous matrix and relatively long distance for electron hopping between the layers.

**Figure 2:**
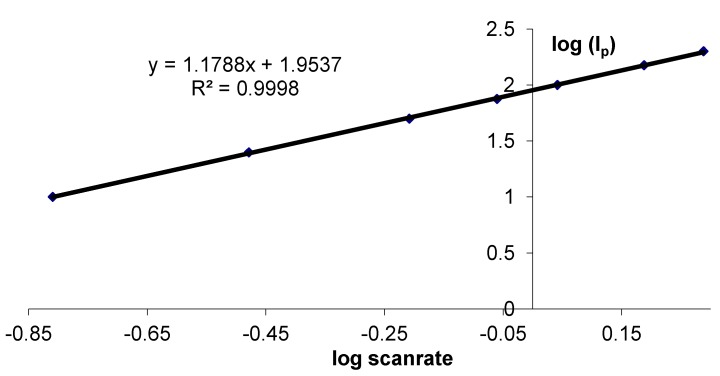
Logarithmic plot between the peak current I_p_ and the scan rate for a spin coated HHC|GelB|GC electrode.

When the HHC containing gelatin layer was immobilized onto the glassy carbon electrode by spin coating, we obtained a much thinner biocompatible layer on top of the electrode surface. Curve 3 is the current-potential behavior of a spin coated (SC) HHC|GelB|GC electrode. This peak shape of oxidation and reduction reactions indicates surface controlled reactions. So, by scaling down, the electrochemical behavior, diffusion controlled reactions evolve to adsorption controlled reactions. To prove this adsorption behavior, logI is plotted *versus* log(scan rate) (Figure 2). The slope of 1.1 indicates the adsorption nature of the process [38].

The cyclic voltammograms of the SC films display approximately symmetric peak shapes and the ratio of the cathodic and anodic peak currents is close to one for all scans. The peak currents and the surface under the peaks (charges) were linearly proportional to all scan rates, indicating less diffusion s and surface-confined electrochemical behavior. The surface coverage of a SC HHC|GelB|GC electrode equals the surface coverage of a DD electrode, *i.e.*, ca. 7 × 10^−11^ mol∙cm^−2^, indicating the fact that only the layers very close to the glassy carbon electrode surface will be detected electrochemically, by recording cyclic voltammetric scans. In this context, it does not make sense to create thick layers of HHC|Gel.

### 3.2. Stability Study of a SC Enzyme-Gelatin Layer

**Figure 3:**
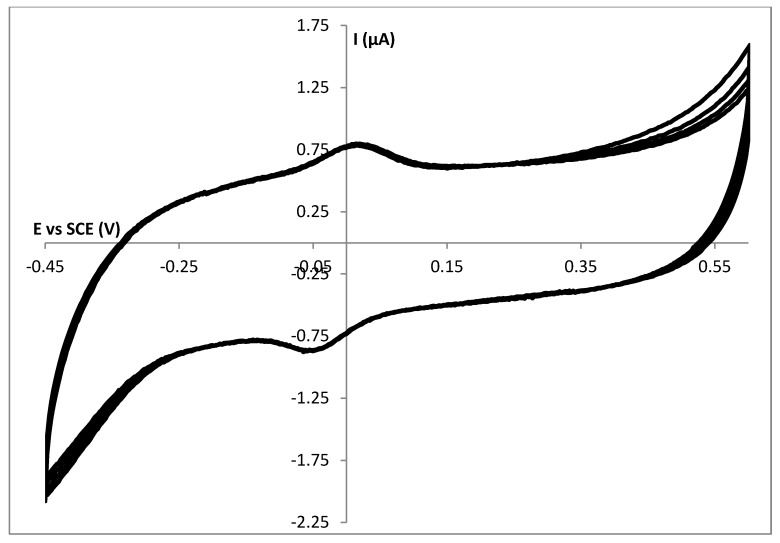
Five successive cyclic voltammograms obtained at a spin coated HHC|GelB|GC electrode in a 10 mmol∙L^−1^ HEPES pH 7 buffer solution with a scan rate of 50 mV∙s^−1^.

When recording successive cyclic voltammograms, we obtained very stable electrochemical behavior meaning that from the first scan all cytochrome *c* molecules can be detected electrochemically. In Figure 3 five successive cyclic voltammograms obtained at a SC HHC|GelB|GC electrode are shown. The gelatin B matrix has an overall negative charge, while HHC is positively charged. Apart from the physical trapping in the hydrogel matrix, this additional interaction further stabilizes the entrapment. 

Compared to the DD layers this is an improvement as these layers are much thicker and time is needed to let the molecules diffuse through the gelatin matrix towards the electrode surface. The molecules have to diffuse to the electrode surface; the gel thickness, degree of cross-linking and the membrane contribute to the mass transport barriers, which the barriers have to overcome before reaching the surface of the electrode [16]. Within the scope of the development of a sensor device, we prefer to have a system that gives its complete electrochemical behavior from the first scan. Another reason to focus on these SC layers is the fact that the amount of electrochemically active DD HHC equals the amount of electrochemically active SC HHC. 

### 3.3. Infrared Characterization of a SC Enzyme-Gelatin Layer

**Figure 4:**
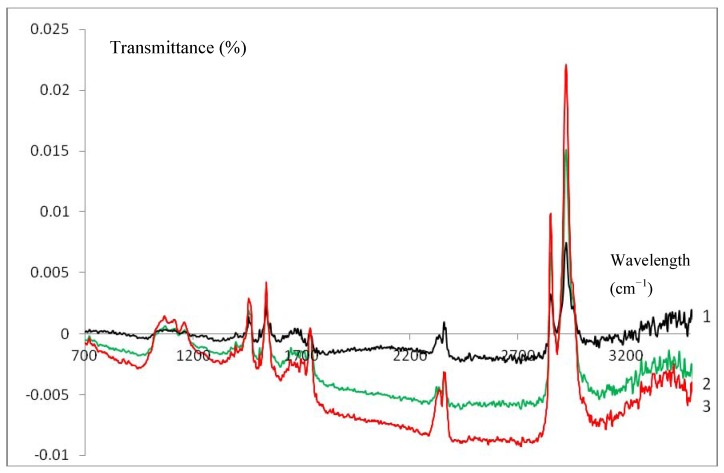
ATR-IR spectra of a spin coated GelB|C (black, 1), Cat|GelB|C (green, 2) and HHC|GelB|C (red, 3) electrode after background correction.

IR measurements were used to give further experimental evidence of the formation of a spin coated gelatin layer. The blank measurement (bare glassy carbon) was subtracted from all recorded IR spectra. Curves 1–3 in Figure 4 are the ATR-IR spectra of a spin coated GelB|GC (black, 1), Cat|GelB|GC (green, 2) and HHC|GelB|GC (red, 3) electrode respectively. In all cases, the characteristic bands for gelatin could be observed [16,18], indicating the presence of the latter on the electrode surfaces. No difference could be made between the gelatin films (1) on top of a glassy carbon electrode and the gelatin enzyme films (2–3). Thus, the presence of enzymes inside the spin coated gelatin film does not affect the spectra. In contrast, the IR spectra of a drop dried HHC|GelB|GC electrode showed distinct differences when compared to a drop dried GelB|GC electrode [16]. This phenomenon could be explained by the fact that for spin coated enzyme-gelatin layers a more homogeneous layer of gelatin (containing enzymes) is attained because of the more forced interaction between both compounds due to the spin coating procedure.

### 3.4. Encapsulation of Cat in a SC Gelatin Film

**Figure 5:**
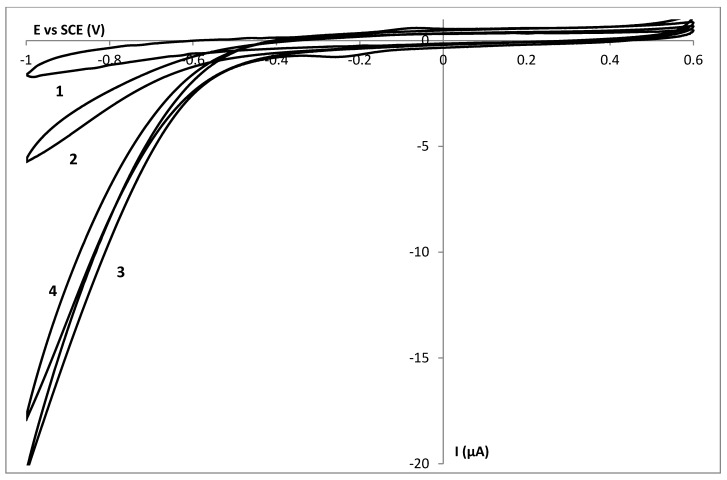
The current potential behavior of a spin coated GelB|C (1–2) and a spin coated Cat|GelB|C (3) electrode in the absence (1) and presence of 10.3 mmol∙L^−1^ H_2_O_2_ (2–3) in a 10 mmol∙L^−1^ HEPES pH 7 buffer solution with a scan rate of 50 mV∙s^−1^. Curve 4 is the current potential behavior obtained after curve 3 and polishing of the electrode.

The electrochemical behavior of a SC gelatin layer on top of a glassy carbon electrode in a HEPES pH 7 buffer solution is shown as curve 1 in Figure 5. No oxidation or reduction processes from gelatin were observed in this specific potential window. When 10.3 mmol∙L^−1^ H_2_O_2_ is added to the solution, curve 2 is obtained, representing the behavior of hydrogen peroxide towards a glassy carbon electrode. 

In a next step, Cat was build into the gelatin spin coated layer and a cyclic voltammetric curve was obtained in the presence of hydrogen peroxide (curve 3). The redox peak of Cat in gelatin without hydrogen peroxide could not be detected electrochemically; this was not a surprise knowing that the immobilization of Cat to an electrode leads to poor electron transfer because the heme group is buried at least 20 Ǻ below the molecular surface [39]. Only by using nanomaterials, can the contact between Cat and the electrode be significantly improved. Based on literature data, we expect Cat to be redox active around −0.47 V *vs*. SCE or at least at this potential we expect to see some electrocatalytic effect when hydrogen peroxide is added [40]. An increase of the reduction current is observed indicating the presence of Cat and its activity towards the target molecules. Unfortunately, after removing the Cat-gelatin layer from the electrode by polishing, curve 4 was obtained. Because an increased reduction current was again observed, it could be stated that Cat was leaking out of the gelatin layer into the solution and some amount of Cat was left in the buffer solution after recording curve 3. To counteract this unwanted phenomenon, gluteraldehyde was successfully added to the gelatin matrix to crosslink the gelatin and the enzyme. 

### 3.5. Thickness Determination

**Figure 6:**
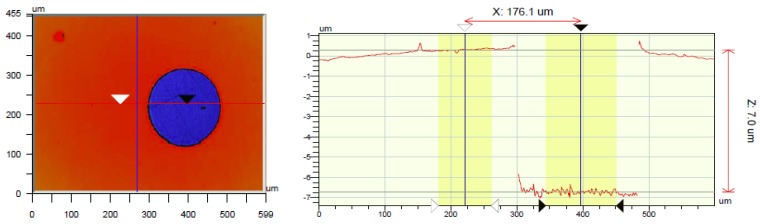
Optical profile measurement showing a gelatin layer thickness of 7 µm. The gelatin was removed locally using an Excimer laser.

To measure the thickness of a SC gelatin layer, a hole was made in the gelatin film with an Excimer laser and the thickness of the film was measured at the edge using a Wyko NT3300 non-contact optical profiler (Figure 6). Following this approach, one avoids the estimation of the index of refraction of the gelatin film needed when measuring through the film. Results show that the average thickness of the Cat-gelatin film is 7 µm.

### 3.6. Characterization of Cat into Gel

**Figure 7:**
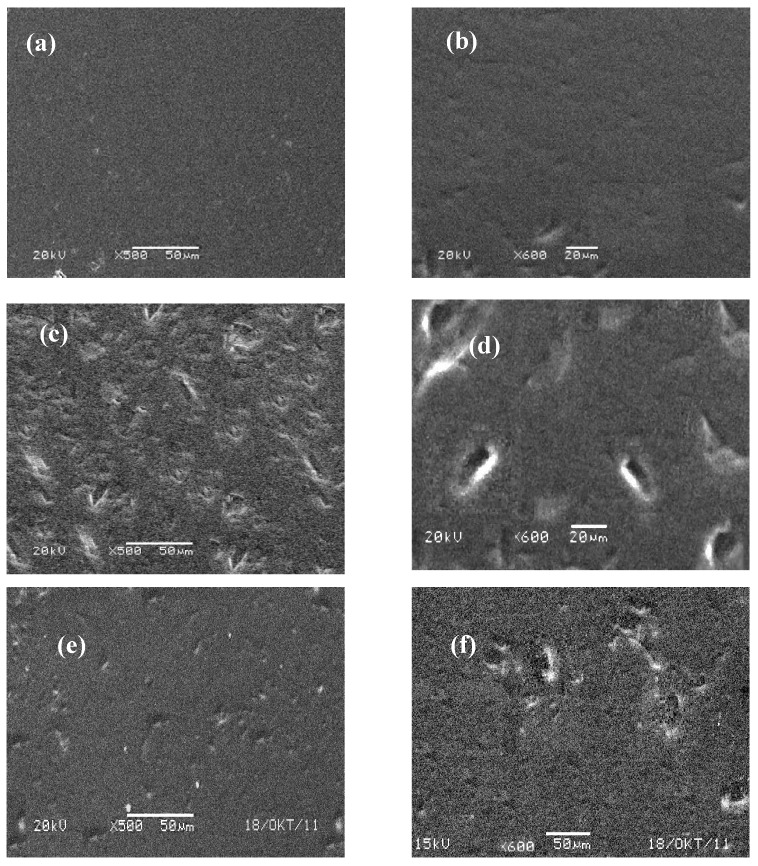
(**a**,**b**) Scanning Electron Microscope (SEM) image of a Gel|C electrode; (**c**,**d**) SEM image of a Cat|Gel|C electrode; (**e**,**f**) SEM image of a Cat|Gel|C electrode which was soaked in a buffer solution for 24 h.

Figure 7 represents the SEM images of the surface of a glassy carbon electrode with pure gelatin (a,b) and catalase entrapped gelatin films (c,d). A significant difference between the surface structure of pure gelatin and Cat/gelatin is observed. In addition, the gelatin film structure seems to be very uniform and regularly flat. In contrast with pure gelatin, as can be seen in Figure 7(c,d), catalase immobilized gelatin layers have an asymmetric and uneven structure pattern which illustrates successful catalase immobilization in a gelatin network. Moreover, this structure change suggests that the interaction between gelatin film and catalase occurs and influences the morphology of the films [28,29].

Figure 7(e,f) demonstrates the morphology of the surface of the catalase immobilized gelatin films that were soaked in buffer solution for 24 h. As shown in the images, there are differences in Cat immobilized films structure in comparison with pure gelatin film but these differences are less in comparison with Cat immobilized film before hanging in the buffer solution which suggests leaching out of the Cat molecules from the gelatin films. Due to charge effects, Cat is leaching out of the gelatin film. In contrast, it has been published previously that HHC stays inside a gelatin film despite the fact that HHC is smaller than Cat.

## 4. Conclusions

In the present study, a hydrogen peroxide biosensor based on catalase immobilized gelatin films was introduced and studied. Cyclic voltammetry was used to verify the surface controlled reactions in the case of spin coated Cat immobilized layers, compared to diffusion controlled reactions for drop dried layers.

FT-IR studies prove spin coated layer formation and demonstrate a strong interaction between the enzyme and the electrode surface which leads to a homogenous structure for these kind of films.

The results show that the thin layer spin coated gelatin layer provides a biocompatible micro environment for Cat immobilization and increases its electroactivity towards the target analyte. It illustrates that these kind of biosensors are promising in the field of development of new electrochemical biosensors.
